# Vaccination with intravenous BCG protects macaques with pre-existing SIV infection from tuberculosis

**DOI:** 10.21203/rs.3.rs-2802306/v1

**Published:** 2023-04-17

**Authors:** Erica C. Larson, Amy L. Ellis-Connell, Mark A. Rodgers, Abigail K. Gubernat, Janelle L. Gleim, Ryan V. Moriarty, Alexis J. Balgeman, Cassaundra L. Ameel, Solomon Jauro, Jaime A. Tomko, Kara B. Kracinovsky, Pauline Maiello, H. Jake Borish, Alexander G. White, Edwin Klein, Allison N. Bucsan, Patricia A. Darrah, Robert A. Seder, Mario Roederer, Philana Ling Lin, JoAnne L. Flynn, Shelby L. O’Connor, Charles A. Scanga

**Affiliations:** 1 Department of Microbiology and Molecular Genetics, University of Pittsburgh, School of Medicine, Pittsburgh, PA, USA.; 2 Center for Vaccine Research, University of Pittsburgh, School of Medicine, Pittsburgh, PA, USA.; 3 Department of Pathology and Laboratory Medicine, University of Wisconsin - Madison, Madison, WI, USA.; 4 Wisconsin National Primate Research Center, University of Wisconsin - Madison, Madison, WI, USA.; 5 Division of Laboratory Animal Resources, School of Medicine, University of Pittsburgh, PA, USA.; 6 Vaccine Research Center, National Institute of Allergy and Infectious Diseases (NIAID), National Institutes of Health (NIH), Bethesda, MD, USA.; 7 Department of Pediatrics, Children’s Hospital of Pittsburgh of the University of Pittsburgh Medical Center, University of Pittsburgh, School of Medicine, Pittsburgh, PA, USA.

## Abstract

Tuberculosis (TB) is the most common cause of death in people living with HIV. BCG delivered intradermally (ID) is the only licensed vaccine to prevent TB. However, it offers little protection from pulmonary TB in adults. Intravenous (IV) BCG, but not ID BCG, confers striking protection against *Mycobacterium tuberculosis* (Mtb) infection and disease in rhesus macaques. We investigated whether IV BCG could protect against TB in macaques with a pre-existing SIV infection. There was a robust influx of airway T cells following IV BCG in both SIV-infected and SIV-naïve animals, with elevated antibody titers in plasma and airways. Following Mtb challenge, all 7 SIV-naïve and 9 out of 12 SIV-infected vaccinated animals were completely protected, without any culturable bacilli in their tissues. PBMC responses post-challenge indicated early clearance of Mtb in vaccinated animals regardless of SIV infection. These data support that IV BCG is immunogenic and efficacious in SIV-infected animals.

## Introduction

*Mycobacterium tuberculosis* (Mtb) infection results in over 10 million cases of active tuberculosis (TB) worldwide every year^1^. People living with HIV (PLWHIV) are very susceptible to Mtb infection and TB disease^2^. TB rapidly progresses and accounts for one in every three deaths among in PLWHIV^3^. Although TB is treatable, complications in PLWHIV such as drug-drug interactions with antiretroviral therapy (ART), issues with compliance due to adverse drug effects, access to healthcare, and development of TB-immune reconstitution inflammatory syndrome (TB-IRIS) remain a barrier to successful TB treatment in this vulnerable population. Vaccines are the best public health approach to prevent infectious diseases like TB, especially in resource-poor settings. However, safety, immunogenicity, and efficacy are a concern in PLWHIV due to immunosuppression from chronic HIV infection.

Bacille Calmette-Guérin (BCG) is the only vaccine currently licensed for TB prevention. BCG is a live-attenuated *M. bovis* strain delivered intradermally at birth. It confers moderate protection against disseminated TB in children (*e.g*. meningeal TB and extrapulmonary TB)^4^, however BCG provides little protection against transmissible pulmonary TB in adolescents and adults, suggesting waning efficacy^5^. The implementation of BCG as a vaccine has remained largely unchanged since its discovery over 100 years ago and it continues to be one of the most widely used vaccines in the world. There is a critical need to improve vaccine efficacy against pulmonary TB in adolescents and adults. However, BCG is contraindicated in people who are immunosuppressed, such as PLWHIV^6^. It is imperative that vaccines are developed that are not only safe and effective for immunocompetent individuals but also protect susceptible populations^4^.

Several groups have investigated different ways to boost protection conferred by BCG, such as subunit vaccines, co-administration with adenoviral vectors encoding Mtb proteins, and use of recombinant strains of BCG^7–10^. However, these efforts were unsuccessful at achieving robust or sterilizing immunity. Studies in mice^11^ and nonhuman primates (NHP)^12^ showed that concurrent infection with Mtb confers protection from a secondary Mtb challenge, suggesting that high-level protection against Mtb is possible. Recently, substantial protection against TB has been achieved in macaque models of TB using several approaches including CMV-vectored delivery of TB antigens^13^ and mucosal delivery of BCG^14^. Recent work by our group and others demonstrated that IV BCG immunization confers robust protection against TB in rhesus^15,16^. Strikingly, IV delivery of BCG protected 9 out of 10 macaques from TB disease (< 100 Mtb CFU), with 6 out of 10 animals exhibiting sterilizing immunity (undetectable CFU)^15^. IV BCG-elicited protection was associated with high levels of mycobacteria-specific T cells in airways, tissue-resident memory T cells^15^ and robust IgM responses^17^.

While the above studies in immunocompetent macaques generated renewed enthusiasm for TB vaccines, these vaccines have not been evaluated in the setting of immune suppression, such as SIV infection. Modeling PLWHIV merits special consideration especially for live attenuated vaccines such as BCG. It remains unknown whether IV delivery of BCG is safe and immunogenic in SIV-infected NHP, and whether the robust protection conferred by IV BCG in healthy animals would be recapitulated in immunocompromised macaques.

Here, we extended the studies of Darrah et al.^15^ to determine whether IV BCG immunization is safe, immunogenic, and protective in our established model of SIV/Mtb coinfection in Mauritian cynomolgus macaques (MCM). MCM have similar high susceptibility to Mtb infection and disease as rhesus macaques^18^ and SIV infection exacerbates TB progression in this species^19^. We show that MCM chronically infected with SIV can be vaccinated with high-dose IV BCG without adverse effects. Furthermore, IV BCG elicits strong immune responses in both SIV-infected and SIV-naïve macaques. MCM responses were consistent with those reported in rhesus macaques^15^, including the induction of T cells in the airways as well as robust antibody responses in blood and airways. Most strikingly, IV BCG conferred robust protection against Mtb challenge irrespective of SIV infection status--providing sterilizing immunity in all 7 SIV-naïve animals and 9 out of 12 SIV-infected animals.

## Results

### IV BCG is safe in SIV-infected animals

**Extended Data Figure 1.**
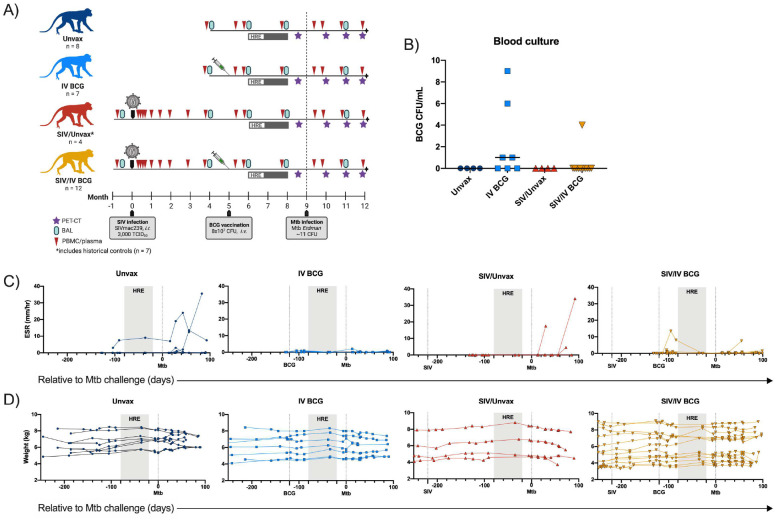
Study outline and clinical parameters. A) Timeline of NHP groups including SIV infection, BCG vaccination, HRE therapy, and Mtb challenge. Sampling schedule of blood draws, BAL, and PET-CT image is also included. B) BCG CFU/mL of blood cultures collected 2 wks after vaccination. Horizontal bars indicate group medians and symbols indicate individual animals (Unvax, n = 4; IV BCG, n = 7; SIV/Unvax, n = 4; SIV/IV BCG, n = 12). C) ESR (mm/hr) of individual animals in each group relative to Mtb challenge. D) Individual animal weights (kg) of each group relative to Mtb challenge. C & D) Symbols indicate individual animals (Unvax, n = 8; IV BCG, n = 7; SIV/Unvax, n = 4; SIV/IV BCG, n = 12).

We sought to determine whether IV BCG was safe, immunogenic, and efficacious in animals with a pre-existing, chronic SIV infection using our established model of SIV/Mtb coinfection in MCM^19,20^. This study included four groups of animals: SIV-naïve, unvaccinated (Unvax) (N=8); SIV-naïve, vaccinated (IV BCG) (N=7); SIV+, unvaccinated (SIV/Unvax) (N=4; historical controls included for TB outcome only, N=7); and SIV+, vaccinated (SIV/IV BCG) (N=12) (Extended Data Figure 1A). For the SIV+ groups, animals were intrarectally infected with SIVmac239 (3,000 TCID_50_). Five months later, some animals were vaccinated intravenously with BCG at 8×10^7^ colony forming units (CFU). Due to concerns over potential disseminated BCG disease in immunocompromised animals, animals were treated with an 8-week regimen of isoniazid/rifampin/ethambutol (HRE) 3–4 weeks after IV BCG vaccination. Unvaccinated animals also received HRE to minimize any confounding effects due to antibiotic therapy. Four weeks after antibiotic cessation (4 months post-BCG in vaccinated animals), all animals were challenged with low-dose Mtb Erdman (~11 CFU) via bronchoscope, followed for 3 months, and then necropsied.

To assess the safety of IV BCG, blood cultures were performed two weeks after vaccination, and erythrocyte sedimentation rate (ESR), an indicator of inflammation, and weight were measured over the course of vaccination. Four of the 7 SIV-naïve, vaccinated animals and 1 of the 12 SIV+ vaccinated animals had culturable BCG in blood two weeks after vaccination (Extended Data Figure 1B). One animal in the SIV+ vaccination group had a transiently increased ESR ~1 month post vaccination (Extended Data Figure 1C). There were no significant changes in clinical status, ESR, or weight in the remainder of the animals following vaccination (Extended Data Figure 1C, D). Thus, based on these clinical measures IV BCG appears to be safe in SIV+ macaques.

**Extended Data Figure 2.**
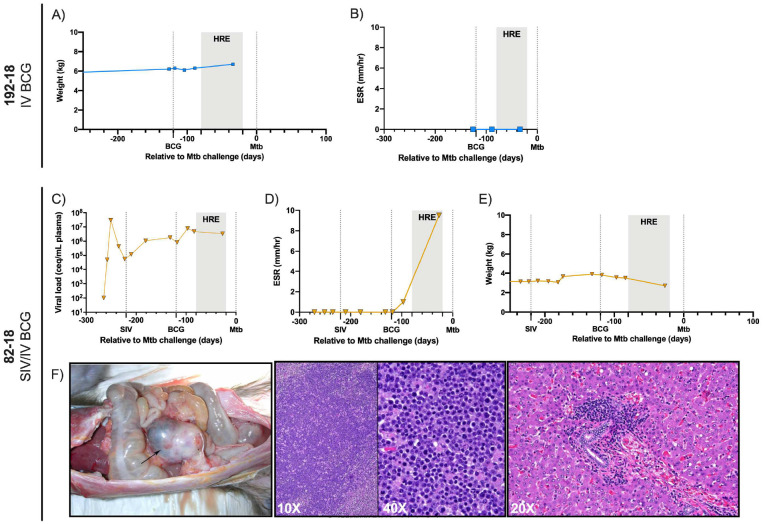
Clinical parameters of 192–18 and 82–18. A) Weight of 192–18 over time relative to Mtb challenge. B) ESR of 192–18 over time. C) Weight of 82–18 over time relative to Mtb challenge. D) ESR of 82–18 over time. E) Plasma viral load over course of study. F) Histopathological signs of lymphoma in 82–18; Left panel: image of ileocecocolic mass (black arrow). Middle panel: 10X (left) and 40X (right) H&E-stained section of ileocecocolic mass indicating large non-cleaved lymphoid population. Right panel: H&E-stained section of liver (20X), peri-portal lymphoid infiltration.

It is worth noting that two IV BCG-vaccinated animals did not reach Mtb challenge, due to reasons unrelated to BCG. The first animal, 192–18, was SIV-naïve and lost 12 weeks after vaccination due to a presumed fatal arrhythmia during BAL with no history of weight loss or elevated ESR (Extended Data Figure 2A, B). The second, 82–18, was SIV-infected and had persistently high plasma viremia (Extended Data Figure 2C). Following IV BCG, it exhibited weight loss and elevated ESR (Extended Data Figure 2D, E). Rapid clinical deterioration ~12 weeks after vaccination led to euthanasia and necropsy where a large abdominal lymphoma was identified (Extended Data Figure 2F). Neither animal had detectable BCG by blood culture two weeks after vaccination and 82–18 showed no histopathological signs of granulomatous disease. Data from these animals were excluded from further analysis.

### IV BCG triggers a transient burst of SIV replication

**Figure 1.**
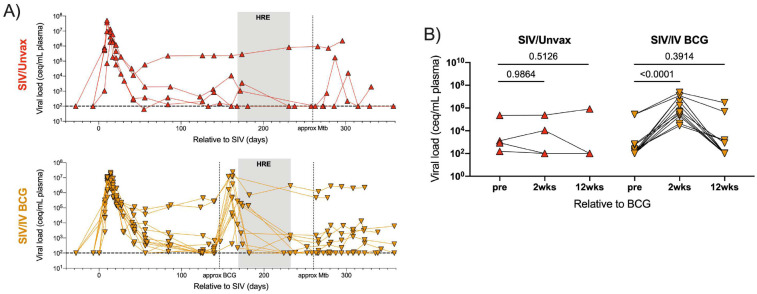
Plasma viremia of SIV+, unvaccinated and vaccinated animals. Plasma viral copy equivalents were determined by qRT-PCR. Each point indicates an individual animal (SIV/Unvax, n = 4; SIV/IV BCG, n = 12). A) Plasma viral load of SIV+, unvaccinated (top panel; red, up-pointing triangles) and SIV+, vaccinated (bottom panel; gold, down-pointing triangles) animals over course of study. Horizontal dashed line represents the limit of detection. B) Plasma viral load of each group prior to (pre), 4 wks, and 12 wks relative to BCG vaccination. Time-matched plasma from SIV+, unvaccinated animals served as a control. Repeated measure one-way ANOVAs were performed on each group. Multiple comparisons relative to ‘pre BCG’ were performed using Dunnett’s multiple comparisons tests. Adjusted p-values are shown.

Plasma viremia was assessed serially in vaccinated and unvaccinated SIV+ animals. All animals in this study had at least one copy of the M1 MHC haplotype. As previously reported^21^, M1+ animals display either spontaneously controlled viremia (<10^4^ copies/mL) or high levels of viremia (>10^5^ copies/mL). As expected, plasma viremia peaked 1–2 weeks post SIV infection and by 7–8 weeks post infection, animals showed a range of viral set points. There was a spike in plasma viremia ~2 weeks following BCG vaccination (Figure 1A). This increase in plasma viremia was significant in SIV+ vaccinated animals compared to time-matched SIV+, unvaccinated animals (Figure 1B). Plasma viremia in SIV+ vaccinated animals returned to their pre-vaccination levels during HRE treatment, prior to Mtb challenge (Figure 1B). Following Mtb challenge, we did not observe a consistent effect on plasma viremia as reported previously^19^. These data indicate that IV BCG was a potent stimulator of viral replication, however this effect was transient.

### IV BCG induces an influx of mycobacterial-specific T cells into airways

**Figure 2.**
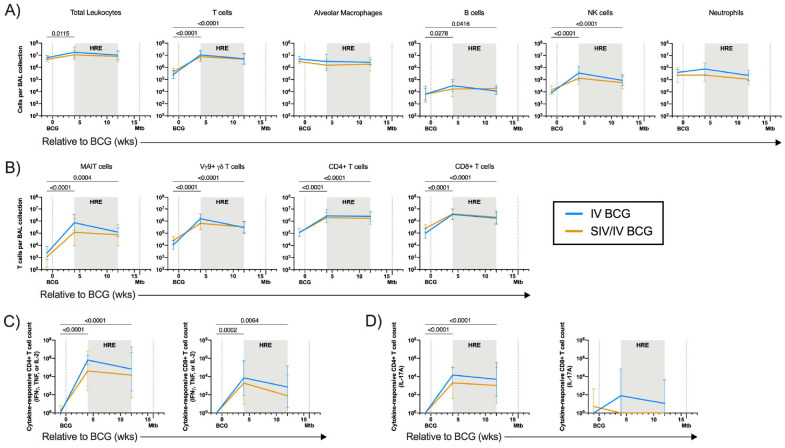
Leukocyte and T cell subsets and T cell response in BAL after BCG vaccination. A) Number of leukocytes per BAL collection. B) Number of T cell subset cells per BAL collection. C) Number of cytokine-responsive (IFNγ, TNF, IL-2) CD4+ and CD8+ T cells in BAL after 14h stimulation with PPD. D) Number of IL-17A-responsive CD4+ and CD8+ T cells in BAL after 14h stimulation with PPD. A-D) Lines indicate mean and error bars indicate SD of SIV-naïve (light blue) and SIV+ (gold) vaccinated animals. SIV-naïve vaccinated animals: pre BCG (n = 5), 4 wks post BCG (n = 6), and 12 wks post BCG (n = 6). SIV+ vaccinated animals: pre BCG (n = 10), 4 wks post BCG (n = 11), and 12 wks post BCG (n = 8). Individual animal data are shown in Supplementary Data 1. Mixed effects models with subject as a random variable were used to assess mean differences among time points and vaccine groups. Time points were compared to ‘pre BCG’ control using Dunnett’s multiple comparison tests. No significant differences between vaccination groups were found. Significant p-values (p < 0.05) across time are shown above each graph. Fixed effect test results and Dunnett’s multiple comparisons are included in Supplementary Table 2A.

Since the primary site of Mtb infection is the lung, we assessed whether IV BCG vaccination led to enhanced immune responses in airways of SIV+ and SIV-naïve MCM as previously described in SIV-naïve rhesus macaques^15^. Flow cytometry on serial bronchoalveolar lavage (BAL) showed a striking influx of T cells into the airways 4 weeks after IV BCG in both SIV-naïve and SIV+ animals (Figure 2A). This high number of T cells was sustained 12 weeks after vaccination (one month prior to Mtb challenge), regardless of SIV status. There was also a significant influx of B cells and NK cells in both vaccinated groups (Figure 2A). MAIT cells, Vγ9+ γδ T cells, CD4+ T cells, and CD8+ T cells significantly increased in number following vaccination and were maintained 12 weeks after vaccination, just prior to Mtb challenge (Figure 2B).

Next, we assessed whether the T cells present in the airways of vaccinated animals were able to produce cytokines associated with Mtb control (IFNγ, TNF, IL-2)^22–24^ in response to mycobacterial-specific antigens (purified protein derivative, PPD). There was a significant increase in the number of cytokine-producing CD4+ T cells, reaching 10^4^-10^6^ cells, in the airways of SIV-naïve and SIV+ vaccinated animals at 4- and 12-weeks after vaccination (Figure 2C). Cytokine-producing CD8+ T cells were significantly elevated, albeit lower than the CD4+ T cells, following vaccination in both BCG-vaccinated groups (Figure 2C). There were increased numbers of mycobacterial-responsive CD4+ T cells producing IL-17A, a cytokine associated with mucosal immunity^25^, in the airways of both SIV-naïve and SIV+ macaques following vaccination (Figure 2D). Mycobacterial-responsive CD8+ T cells producing IL-17A was incredibly low in both groups (Figure 2D). Taken together, these data indicate that IV BCG vaccination induces a rapid and sustained influx of mycobacterial-specific T cells into the airways, regardless of SIV status.

### IV BCG induces mycobacterial-specific responses in PBMC

**Figure 3.**
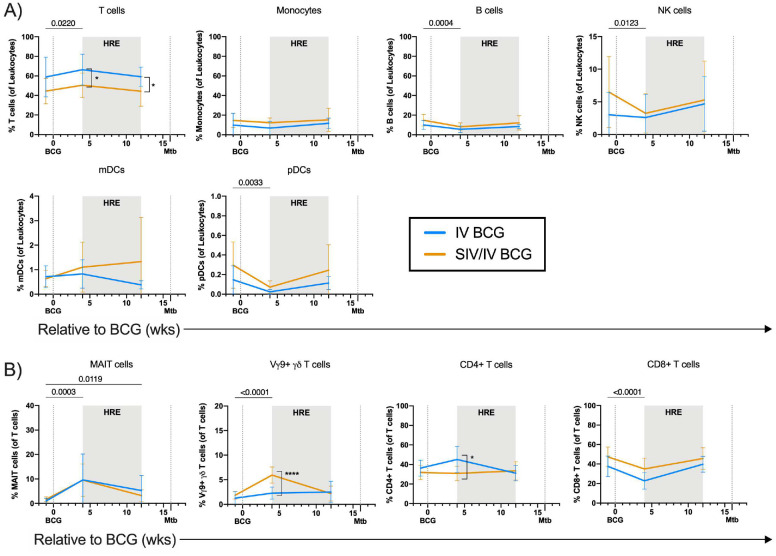
Frequencies of Leukocyte and T cell subsets in PBMC after vaccination. A) Frequencies of leukocyte subsets relative to BCG. B) Frequencies of T cells and T cell subsets (MAIT, Vγ9+, CD4+, and CD8+ T cells) relative to BCG. Lines indicate mean and error bars indicate SD of SIV-naïve (IV BCG; light blue) and SIV+ (SIV/IV BCG; gold) vaccinated animals. SIV-naïve vaccinated animals: pre BCG (n = 7), 4 wks post BCG (n = 7), and 12 wks post BCG (n = 7). SIV+ vaccinated animals: pre BCG (n = 12), 4 wks post BCG (n = 12), and 12 wks post BCG (n = 11). Individual animal data are shown in Supplementary Data 2. Mixed effects models with subject as a random variable were used to assess mean differences among time points and vaccine groups. Time points were compared to ‘pre BCG’ control using Dunnett’s multiple comparison tests. Significant p-values (p < 0.05) across time are shown above each graph. Significant differences determined by Mann-Whitney tests between vaccination groups at given time points are indicated by brackets and significant p-values (not adjusted for multiple comparison) indicated: *p < 0.05 and ****p < 0.0001. Fixed effect test results, Dunnett’s multiple comparisons, and Mann-Whitney test results are reported in Supplementary Table 2B.

We assessed the circulating immune cell landscape and T cell responses to mycobacterial and Mtb-specific stimuli in PBMC. There was a modest but significant increase in the frequency of T cells, along with a small decline in B cell, NK cell, and plasmacytoid dendritic cell (pDC) populations, following BCG vaccination at 4 weeks (Figure 3A). The frequency of MAIT cells significantly increased 4 weeks after vaccination and were maintained 12 weeks after vaccination, regardless of SIV status (Figure 3B). Vγ9+ γδ T cells significantly increased 4 weeks after BCG vaccination in both vaccinated groups (Figure 3B). However, SIV+ animals had significantly higher frequencies of Vγ9+ γδ T cells compared to SIV-naïve animals (Figure 3B). Vγ9+ γδ T cell frequencies returned to pre-infection levels by 12 weeks post-BCG. The frequency of CD4+ T cells did not change significantly over vaccination (Figure 3B), although, CD4+ T cell frequencies were significantly lower in SIV+ compared to SIV-naïve animals 4 weeks after IV BCG (Figure 3B), most likely due to the transient spike in SIV replication^26^. CD8+ T cells, on the other hand, significantly declined in both vaccinated groups 4 weeks after BCG vaccination (Figure 3B). Frequencies of both CD4+ and CD8+ T cells returned to near pre-infection baseline levels by week 12 after IV BCG.

**Extended Data Figure 3.**
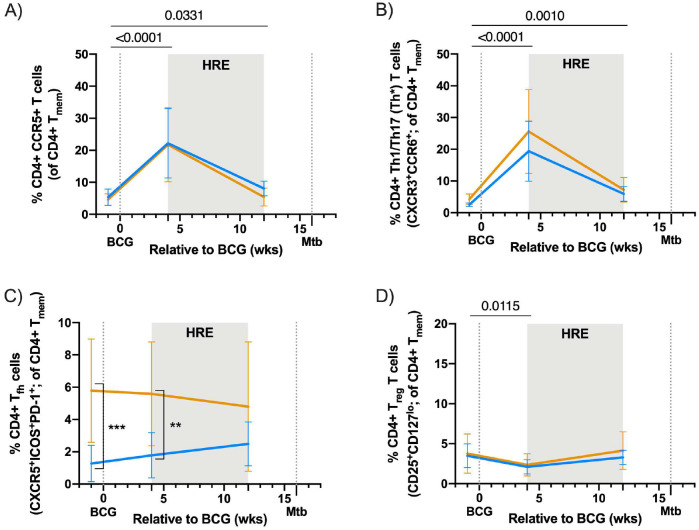
CD4+ T cell phenotype in PBMC after vaccination. A) Frequency of CD4+ CCR5+ T cells in PBMC relative to BCG vaccination. B) Frequency of CD4+ Th1/Th17 (Th*) T cells (CXCR3^+^CCR6^+^) in PBMC relative to BCG vaccination. C) Frequency of CD4+ T_fh_ T cells (CXCR5^+^ICOS^+^PD-1^+^) in PBMC relative to BCG vaccination. D) Frequency of CD4+ T_reg_ T cells (CD25^+^CD127^lo^) in PBMC relative to BCG vaccination. A-D) Lines indicate mean and error bars indicate SD of SIV-naïve (light blue) and SIV+ (gold) vaccinated animals. SIV-naïve vaccinated animals: pre BCG (n = 7), 4 wks post BCG (n = 7), and 12 wks post BCG (n = 7). SIV+ vaccinated animals: pre BCG (n = 12), 4 wks post BCG (n = 12), and 12 wks post BCG (n = 11). Individual animal data are shown in Supplementary Data 3. Linear mixed effects models with subject as a random variable were used. Fixed effect tests were used to assess mean differences among time points and vaccine groups. Time points were compared to ‘pre BCG’ control using Dunnett’s multiple comparison tests. Significant p-values (p < 0.05) across time are shown above each graph. Significant differences determined by Mann-Whitney tests between vaccination groups at given time points are indicated by brackets and significant p-values indicated: **p < 0.01, ***p < 0.001, and ****p < 0.0001. Fixed effect test results, Dunnett’s multiple comparisons, and Mann-Whitney test results are reported in Supplementary Table 2C.

CCR5+ and CXCR3+CCR6+ CD4+ T cells frequencies peaked in PBMC 4 weeks after vaccination and remained elevated compared to pre-BCG regardless of SIV status (Extended Data Figure 3A, B). CCR5 is expressed on effector T cells and is a co-receptor the facilitates HIV/SIV infection^27,28^, whereas T cells expressing both CXCR3 and CCR6 are associated with Th1/Th17 (Th1*) responses^29,30^. In addition, the frequency of circulating CD4+ T follicular helper (T_fh_) cells in SIV+ vaccinated animals were elevated across all time points, including pre-BCG, compared to SIV-naïve animals likely due to chronic SIV (Extended Data Figure 3C). There was a small but significant reduction in CD4+ T regulatory (T_reg_) cells in both vaccinated groups 4 weeks after IV BCG (Extended Data Figure 3D).

**Figure 4.**
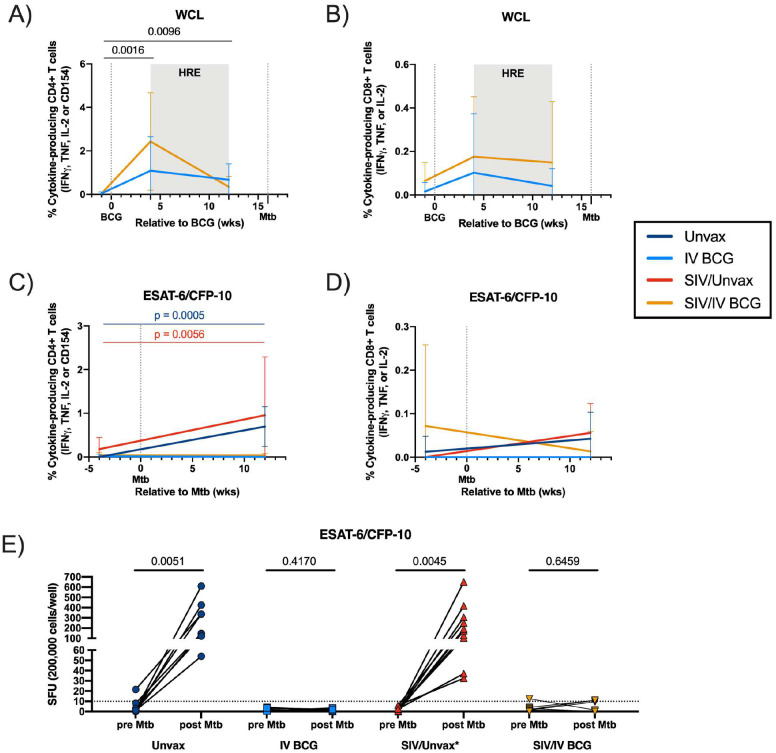
T cell response in PBMC after BCG vaccination and Mtb challenge. A & B) Frequency of cytokine-responsive CD4+ (A) and CD8+ (B) T cells in PBMC after 14h stimulation with H37Rv whole cell lysate relative to BCG vaccination. Mixed effects models with subject as a random variable were used to assess mean differences among time points and vaccine groups. Time points were compared to ‘pre BCG’ control using Dunnett’s multiple comparison tests. Significant p-values (p < 0.05) across time are shown above each graph. Fixed effect test results and Dunnett’s multiple comparisons are reported in Supplementary Table 2D. C & D) Frequency of cytokine-responsive CD4+ (C) and CD8+ (D) T cells in PBMC after 14h stimulation with ESAT-6/CFP-10 relative to Mtb challenge. Linear mixed effects models were used to determine significant mean differences between time and animal group. Šidák’s multiple comparisons test p-values are reported in Supplementary Table 2D. Significant p-values (p < 0.05) across time are shown above each graph. Mean differences between groups are indicated by colored brackets corresponding to significantly different groups (Unvax, dark blue; SIV/Unvax, red) and significant p-values are indicated: **p < 0.01, ***p < 0.001. A-D) Lines indicate mean and error bars indicate SD of Unvax (dark blue), IV BCG (light blue), SIV/Unvax (red) and SIV/IV BCG (gold) animals. Unvaccinated animals (Unvax): Pre Mtb (n = 8) and 12 wks post Mtb (n = 8). SIV-naïve vaccinated animals (IV BCG): pre BCG (n = 7), 4 wks post BCG (n = 7), and 12 wks post BCG/Pre Mtb (n = 7), and 12 wks post Mtb (n = 7). SIV+ unvaccinated (SIV/Unvax): Pre Mtb (n = 4) and 12 wks post Mtb (n = 4). SIV+ vaccinated animals (SIV/IV BCG): pre BCG (n = 12), 4 wks post BCG (n = 12), 12 wks post BCG/Pre Mtb (n = 11), and 12 wks post Mtb (n = 12). Individual animal data are shown in Supplementary Data 4. E) IFNγ production in PBMC prior to and after Mtb challenge by ELISpot. Paired t tests were used to determine significance within each group. P-values are shown.

CD4+ and CD8+ T cells in blood produced cytokines (IFNγ, TNF, IL-2, CD154) upon stimulation to mycobacterial antigens (Mtb whole cell lysate (WCL)) at 4 and 12 weeks post-BCG (Figure 4A, B), with no significant differences between SIV-naïve and SIV+ macaques. However, following Mtb challenge, cytokine production in response to peptide pools of the Mtb-specific antigens, ESAT-6 and CFP-10 (antigens not present in BCG), by CD4+ T cells was only observed in non-vaccinated SIV-naïve or SIV+ macaques (Figure 4C, D). IFNγ ELISpot assays of PBMC collected pre- and post-Mtb challenge also showed that both unvaccinated groups generated an IFNγ response to Mtb-specific ESAT-6 and CFP-10 (Figure 4E). Strikingly, PBMC from vaccinated animals, regardless of SIV status, did not generate an IFNγ response to Mtb antigens. These data suggest that IV BCG induces rapid and early clearance of Mtb precluding a measurable T cell response to infection, as noted in our previously published study on IV BCG in SIV-naïve rhesus macaques^15^.

### IV BCG induces mycobacterial-specific IgG, IgA, and IgM in blood and airways

**Figure 5.**
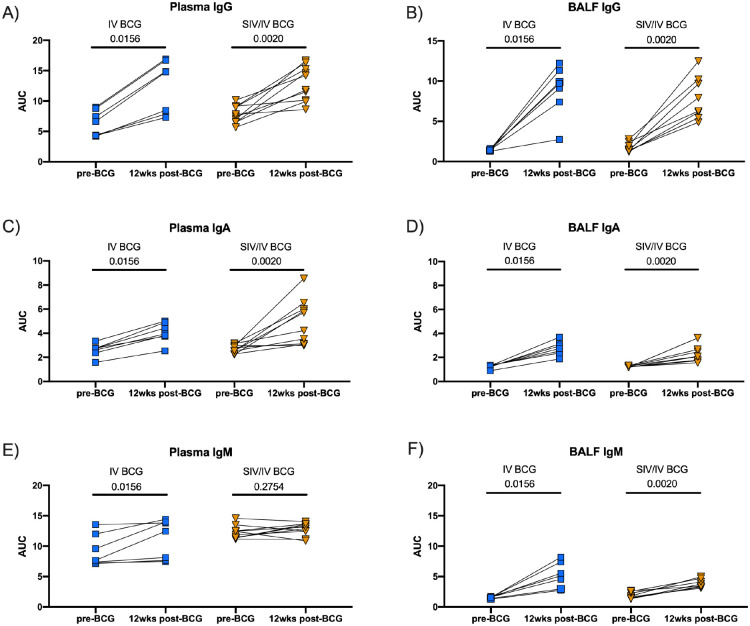
Mycobacterial-specific antibodies in plasma and BALF following BCG vaccination. Antibodies specific for H37Rv whole cell lysate were assessed by ELISA for both vaccinated groups. A) Plasma IgG. B) BALF IgG. C) Plasma IgA. D) BALF IgA. E) Plasma IgM. F) BALF IgM. A-F) Area Under the Curve (AUC) was determined by calculating the area under the dilution series curve using GraphPad Prism. Individual animals indicated by symbols for IV BCG (light blue square; n = 7) and SIV/IV BCG (gold, down-pointing triangle; n = 12). Wilcoxon paired signed rank tests were performed to determine significance. P-values are shown.

Recent studies have shown that humoral immunity may play role in the control of Mtb infection and was associated with protection by IV BCG^17,31–33^. Humoral responses to mycobacterial antigens (WCL) in plasma and BAL fluid (BALF) prior to and 12 weeks after IV BCG vaccination (4 weeks prior to Mtb challenge) were assessed by ELISA. There were significant increases in WCL-reactive IgG and IgA levels in the plasma and BALF of both SIV-naïve and SIV+ animals following vaccination (Figure 5A–D). WCL-reactive IgM in plasma increased after vaccination in SIV-naïve but not in SIV+ animals (Figure 5E). It is possible that we were unable to detect a change in plasma IgM in the SIV+ animals due to the high baseline IgM prior to IV BCG vaccination. Nevertheless, there was a significant increase in BALF IgM levels in both groups after IV BCG vaccination (Figure 5F). Thus, IV BCG induces mycobacterial-specific humoral immune responses in both SIV-naïve and SIV+ animals.

### IV BCG protects SIV+ macaques from TB

**Figure 6.**
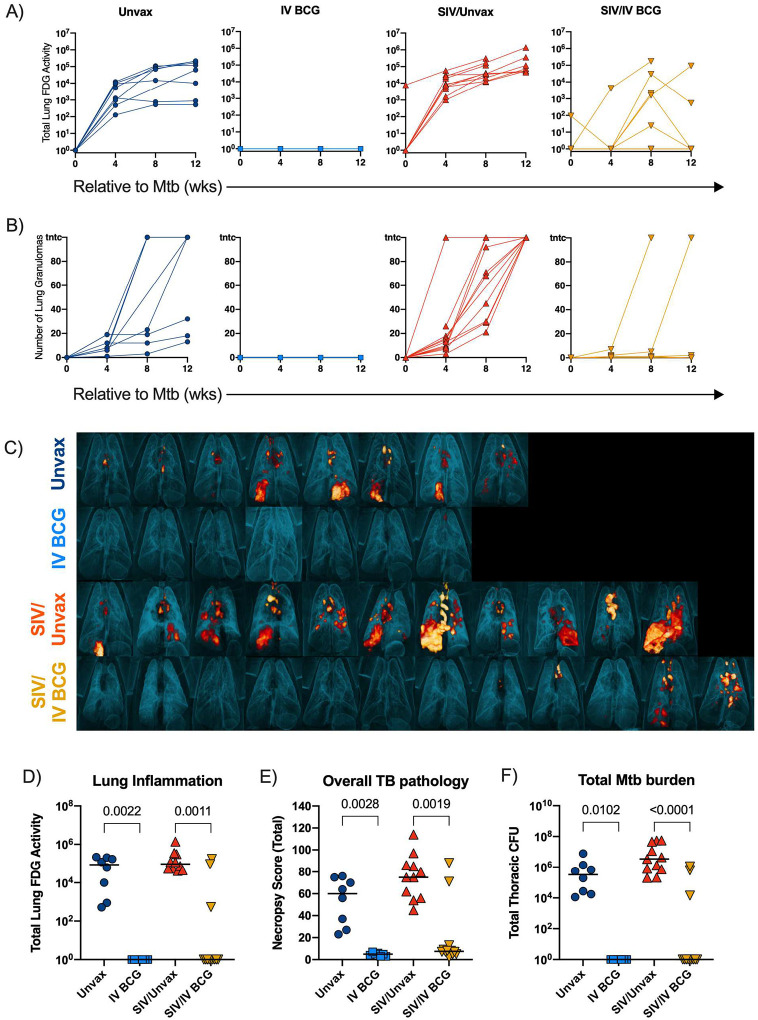
Protection against Mtb challenge in BCG vaccinated groups. A) Total FDG activity (lung inflammation) relative to Mtb challenge, measured by PET/CT imaging. Lines indicate individual animals. B) Number of lung granulomas relative to Mtb challenge. At 4 & 8 wks, granulomas were counted by CT, whereas at 12 wks, granulomas were counted by gross pathology. C) Three-dimensional renderings of PET/CT images of individual animals taken at necropsy. D-F) Lung inflammation (D), overall TB pathology (E), and total Mtb burden (thoracic CFU) (F) at necropsy. Each point indicates an individual animal and horizontal bars indicate group medians (Unvax, n = 8; IV BCG, n = 7; SIV/Unvax, n = 11; SIV/IV BCG, n = 12). Kruskal Wallis tests were performed with Dunn’s multiple comparisons between SIV-naïve, vaccinated and unvaccinated groups (dark blue circles and light blue squares, respectively); and SIV+, vaccinated and unvaccinated groups (red, up-pointing triangle and gold, down-pointing triangle, respectively). P-values are shown.

Four months after BCG vaccination and one month after HRE cessation, animals were challenged with low dose Mtb Erdman via bronchoscope. Inflammation and progression of Mtb infection were serially quantified using ^18^F-fluorodeoxyglucose (FDG) positron emission tomography-computed tomography (PET/CT) imaging. Total lung FDG activity, a surrogate for lung inflammation, revealed substantial lung inflammation in both unvaccinated groups as early as 4 weeks post-Mtb challenge, which remained elevated until necropsy (Figure 6A). In contrast, SIV-naïve vaccinated animals displayed minimal lung inflammation over the 12 weeks of Mtb infection (Figure 6A). SIV+ vaccinated animals showed variable lung inflammation, especially at 8 weeks post-Mtb challenge (Figure 6A). We previously showed that dissemination of granulomas, an indicator of TB disease progression, occurred between 4 and 8 weeks after Mtb coinfection in SIV+ animals^19^. We observed a similar effect here (Figure 6B). IV BCG vaccination of SIV-naïve MCMs resulted in minimal granuloma formation and no apparent dissemination, recapitulating the results from our prior study in rhesus macaques^15^ (Figure 6B). Remarkably, most SIV+ vaccinated macaques also had minimal granuloma formation and dissemination, although two animals did show progressive disease by this metric (Figure 6B). PET/CT imaging prior to necropsy revealed striking differences in TB disease between the unvaccinated and vaccinated animals (Figure 6C). Both SIV-naïve and SIV+ vaccinated animals had significantly less lung inflammation compared to their respective unvaccinated controls at necropsy, although three of the 12 SIV+ vaccinated animals did have increased lung FDG activity (Figure 6D).

**Extended Data Figure 4.**
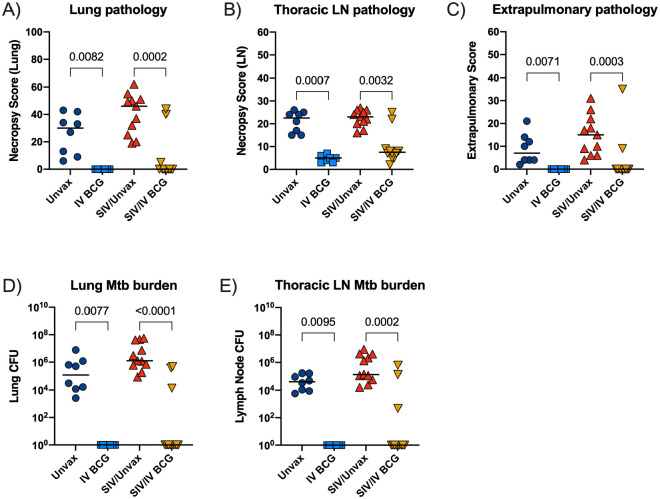
TB outcome by tissue compartment. A-E) Lung pathology (A), thoracic lymph node (LN) pathology (B), extrapulmonary pathology (C), lung Mtb burden (D), and thoracic LN Mtb burden (E) at necropsy. Each point indicates an individual animal and horizontal bars indicate group medians (Unvax, n = 8; IV BCG, n = 7; SIV/Unvax, n = 4; SIV/IV BCG, n = 12). Kruskal Wallis tests were performed with Dunn’s multiple comparisons between SIV-naïve, vaccinated and unvaccinated groups (dark blue circles and light blue squares, respectively); and SIV+, vaccinated and unvaccinated groups (red, up-pointing triangle and gold, down-pointing triangle, respectively). P-values are shown.

TB pathology was assessed at necropsy using an established scoring system^18^. IV BCG resulted in a significant reduction in lung pathology regardless of SIV, although there were two SIV+ vaccinated animals with high pathology scores (Figure 6E). Multiple tissue samples were plated at time of necropsy and Mtb bacterial burden (CFU) was quantified. Tissues isolated from all 7 SIV-naïve vaccinated animals and 9 of 12 SIV+ vaccinated animals were completely sterile 12 weeks after Mtb challenge (Figure 6F). IV BCG vaccination resulted in a significant reduction of pathology and Mtb burden across different tissue compartments (lung, thoracic lymph node, and extrapulmonary sites), regardless of SIV status (Extended Data Figure 4A–E). With protection from TB defined as a total thoracic Mtb burden <100 CFU^15^, IV BCG conferred 100% protection in the SIV-naïve animals (7/7) and 75% protection in the SIV+ animals (9/12).

**Extended Data Figure 5.**
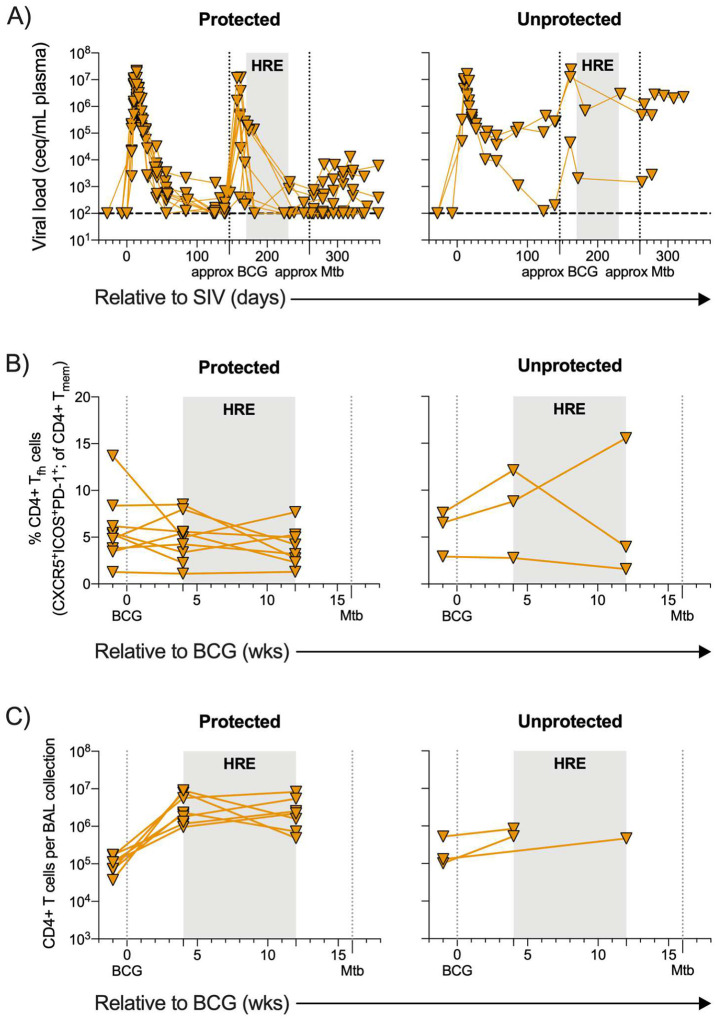
Plasma viral load, PBMC CD4+ Tfh T cells, and BAL CD4+ T cell levels stratified by protection across SIV+, vaccinated animals. Individual symbols and lines indicate protected animals (thoracic CFU < 100, n = 9) and unprotected animals (thoracic CFU > 100, n = 3). A) Plasma viremia in protected and unprotected SIV+, vaccinated animals over the course of the study. B) Frequency of CD4+ T_fh_ T cells (CXCR5^+^ICOS^+^PD-1^+^) in PBMC from protected and unprotected SIV+, vaccinated animals relative to BCG vaccination. C) Number of CD4+ T cells per BAL collection in protected and unprotected SIV+, vaccinated animals relative to BCG vaccination.

The three unprotected SIV+ vaccinated animals tended to have higher plasma viremia levels (Extended Data Figure 5A). Moreover, these three animals appeared to have elevated levels of circulating CD4+ T_fh_ cells and fewer CD4+ T cells in the airways over IV BCG vaccination (Extended Data Figure 5B, C). These data suggest that at least 2 of the unprotected animals showed signs of progressive SIV infection. Progressive SIV impairs immunity, especially through the depletion of CD4+ T cells^26,34^. The high viral replication in these animals may have compromised the ability of IV BCG to elicit a successful anti-mycobacterial response, resulting in poor protection against Mtb.

Overall, these data demonstrate that IV BCG vaccination resulted in complete protection against Mtb in SIV-naïve animals. Furthermore, we show for the first time that high-order protection against Mtb is achievable in SIV+ animals.

## Discussion

There is an enormous need for an effective vaccine against TB in PLWHIV. Here we show the remarkable result that IV BCG can confer sterilizing immunity in SIV+ macaques. In addition, we recapitulated our previously published robust protection and sterilizing immunity provided by IV BCG in SIV-naïve rhesus macaques^15^ in a different SIV-naïve macaque species, MCM. Immune responses to IV BCG were similar in SIV-naïve and SIV+ macaques, with a robust and sustained expansion of mycobacterial-specific CD4+ and CD8+ T cells in the airways, lower but significant increases in γδ T cells and MAIT cells and enhanced mycobacterial-reactive antibody responses in blood and airways.

To date, BCG is contraindicated in PLWHIV due to the possibility of disseminated BCG disease^5^. Previous studies in humans and NHP have detected BCG in inoculation sites after vaccination^15,35–37^. In the current study, there were no signs of disseminated BCG disease following vaccination of SIV+ MCM, including the SIV+ IV BCG animal necropsied before Mtb challenge. The 9 SIV+ IV BCG-vaccinated animals that were protected had completely sterile tissues at necropsy, with no culturable Mtb (or BCG) present. Moreover, SIV+ animals had negative blood cultures after IV BCG and stable ESRs and body weight, indicating high-dose (8 × 10^7^ CFU) IV BCG was well-tolerated. It is important to note that the vaccinated animals were treated with antimycobacterial drugs beginning within 4 weeks of vaccination as an added precaution. Yet the IV BCG-induced immune responses and protection were not diminished in all SIV-naïve macaques and in most SIV+ macaques, suggesting that BCG can be killed soon after vaccination without loss of protection.

We noted a transient spike in plasma viremia 2 weeks after IV BCG vaccination, which was mirrored by an increased frequency of circulating CCR5+ CD4+ T cells two weeks later. BCG vaccination has been shown to increase these cells in HIV-exposed infants and infant macaques^38,39^. Given that CCR5+ CD4+ T cells are a target of SIV^28^, the observed spike in viremia is likely due to the expansion of infectable cells induced by BCG. The increase of both CCR5+ CD4+ T cells and plasma viremia was transient, declining after 4 weeks to pre-vaccination levels. Nonetheless, a transient burst of SIV replication following IV BCG may increase the viral reservoirs in tissues and represent a safety signal for TB vaccines in the context of HIV infection. However, this effect may be mitigated by concurrent ART.

HIV infection is known to impair immune responses. One concern of concurrent HIV infection is whether it will interfere with IV BCG-elicited immune responses and reduce vaccine efficacy in PLWHIV^40^. We previously reported an influx of mycobacterial-specific CD4+ and CD8+ T cells in the airways of IV BCG-vaccinated SIV-naïve rhesus macaques^15^. Here, we observed a similar influx of airway CD4+ and CD8+ T cells producing IFNγ, TNF, and/or IL-2 in SIV+, as well as SIV-naïve, animals 4 weeks after IV BCG vaccination. Mycobacterial-specific CD4+ T cells in PBMC also increased after vaccination in both SIV+ and SIV-naïve animals. Dijkman et al. showed that animals vaccinated mucosally with BCG generated polyfunctional Th17 cells in airways, and these cells correlated with protection^14^. T1/T17 CD4+ and CD8+ T cells were associated with lower bacterial burden in granulomas, indicative of their role in Mtb control^41^. We observed here an influx of IL-17A-producing CD4+ T cells, but not CD8+ T cells, in airways and an increase in circulating Th1/Th17 (Th1*) CD4+ T cell frequencies in both SIV+ and SIV-naïve groups after IV BCG vaccination. Antibodies may play a role in Mtb control as well^42^. We demonstrated that mycobacterial-specific antibodies increased in plasma and airways following IV BCG vaccination in both SIV+ and SIV-naïve animals. Collectively, these data indicate that SIV infection did not drastically impair the generation of mycobacterial-specific T cell or antibody responses in airways and blood. Furthermore, these responses were maintained despite anti-mycobacterial drug treatment initiated by 4 weeks after administration of IV BCG. BCG has been shown to enhance innate immunity against TB through epigenetic modifications of hematopoietic stem cells, referred to as trained immunity^43–46^. Although this was not investigated in the current study, trained immunity in the context of pre-existing SIV and BCG vaccination will be a focus in future studies.

Several recent studies have demonstrated high-order protection against TB elicited by a vaccine. Hansen et al. reported 14 of 38 rhesus macaques vaccinated with the viral vector RhCMV/TB showed no sign of disease after Mtb challenge^13^. Dijkman et al. showed that mucosal delivery of BCG was able to protect from TB in a repeated, low-dose Mtb challenge model^14^. We explored different routes of BCG vaccination in SIV-naïve rhesus macaques and concluded that IV delivery was the most protective route^15^. Here we show a similar degree of protection elicited by IV BCG in MCM. All 7 SIV-naïve animals were protected from Mtb challenge. Most strikingly, 75% (9 of 12) of SIV+ animals were completely protected from Mtb challenge by IV BCG, indicating that IV BCG vaccination can generate protective immunity in SIV+ animals.

Three SIV+ vaccinated animals were unprotected from Mtb challenge. These animals tended to have a higher viral setpoint, more circulating T_fh_, and fewer CD4+ T cells in BAL, suggesting that unprotected animals had progressive SIV infections^26,47,48^. In a study of BCG coinfection of SIV+ rhesus macaques, animals with high plasma viremia (>10^6^ copies/mL) exhibited fewer circulating CD4+ T cells and disseminated BCG^37^. Airways were not sampled in that study. While we did not observe disseminated BCG in the 3 unprotected animals, it is possible that the progressive SIV infection ablated the development of an effective anti-mycobacterial T cell response elicited by IV BCG. Conversely, the SIV+ animals that were protected by IV BCG tended to have better-controlled SIV and more CD4+ T cells in the airways. Viremic control, achieved either naturally or by ART, may be required to ensure IV BCG-elicited protection in SIV+ animals and will be explored in future studies.

IV BCG has many hurdles to clear before it could be translated to the clinic. Importantly, vaccine-elicited protection was observed with just 3–4 weeks of BCG exposure prior to anti-BCG therapy initiated to minimize the risk of disseminated BCG. Treating vaccinees with 8 weeks of anti-BCG drugs is obviously infeasible and it is not yet known precisely how long BCG needs to be alive to elicit protection. A self-limiting auxotrophic BCG strain may represent a safe and effective alternative without the need for a post vaccination antibiotic regimen^49^. The IV administration route have proven successful for infectious diseases like malaria^50^. Yet, IV administration of a live bacteria, such as BCG, poses challenges to clinical translation. Recent studies in SIV-naïve rhesus macaques have shown promising results with mucosally delivered BCG^14,51^ or MTBVAC^44,52^ and merit exploration in SIV-infected animals. Overall, this study is the first to show robust vaccine-elicited protection against Mtb infection and disease using a NHP model of HIV. Furthermore, it establishes a model to identify correlates of protection in the context of pre-existing SIV/HIV and lays the groundwork for future studies to develop more easily translatable vaccine regimens.

## Online Methods (Nature submissions)

### Animals

Adult Mauritian cynomolgus macaques (*Macaca fascicularis*; age > 4 years old) were obtained from Bioculture US (Immokalee, FL). MHC haplotype was determined by MiSeq sequencing and animals with the presence of at least one copy of the M1 MHC haplotype were selected for this study^53,54^.

Animal protocols and procedures were approved by the University of Pittsburgh Institutional Animal Care and Use Committee (IACUC) which adheres to guidelines established in the Animal Welfare Act and the Guide for the Care and Use of Laboratory Animals, as well as the Weatherall Report (8th Edition). The University is fully accredited by AAALAC (accreditation number 000496), and its OLAW animal welfare assurance number is D16–00118. The IACUC reviewed and approved the study protocols 15035407 and 18032418, under Assurance Number A3187–01 and D16–00118, respectively.

Animal welfare was monitored twice daily for overall physical health (weight, appetite, activity level, etc.) as described previously^20^. Animals were monitored closely following Mtb challenge for clinical signs of TB (*e.g*., weight loss, tachypnea, dyspnea, or coughing). In addition, regular PET/CT imaging was conducted to monitor TB progression. Animals were sedated for all veterinary procedures (*e.g*., blood draws) using ketamine or other approved drugs. Veterinary technicians monitored animals especially closely for any signs of pain or distress, and provided appropriate supportive care (*e.g*., dietary supplementation and rehydration) and treatments (analgesics) when necessary. Any animal considered to have advanced disease or intractable pain from any cause, was deemed to have reached the humane endpoint, sedated with ketamine and humanely euthanized using sodium pentobarbital.

### SIV infection

Vaccinated and unvaccinated macaques designated for SIV infection were infected intrarectally with SIVmac239 (3,000 TCID50 IU). Plasma viremia was monitored serially by qPCR as previous described^55,56^.

### BCG vaccination

BCG Danish Strain 1331 (Statens Serum Institute, Copehagen, Denmark) was expanded (by Aeras, now AVI) and frozen at approximately, 3 × 10^8^ CFU/mL. Just prior to BCG injection, BCG was thawed and diluted in sterile PBS to approximately 5 × 10^7^ CFU/mL. IV BCG was injected into the left saphenous vein in a volume of 1 mL. An aliquot of inoculum was plated on 7H11 agar and CFU was enumerated 3 weeks later to ensure an accurate input inoculum.

### Mtb challenge

All animals were infected with a low dose (4 – 21 CFU) of Mtb Erdman via bronchoscopic instillation, as described previously^18^.

### Clinical and microbiological monitoring

All animals were assessed twice daily for general health over the entirety of the study. Blood cultures were performed two weeks after IV BCG to assess BCG CFU/mL. Blood was plated on two 7H11 plates at 500 μL per plate, incubated for 21 days, and colonies were enumerated. Following Mtb challenge, animals were monitored closely for clinical signs of TB (coughing, weight loss, tachypnea, dyspnea, etc.) following Mtb challenge. Monthly gastric aspirates (GA) and bronchoalveolar lavage (BAL) samples were tested for Mtb growth. Blood was drawn at regular intervals to measure erythrocyte sedimentation rate and to provide peripheral blood mononuclear cells (PBMC) and plasma.

### PBMC and BAL processing

PBMC were isolated from blood using Ficoll-Paque PLUS gradient separation (GE Healthcare Biosciences. Single-cell suspensions were cryopreserved in FBS containing 10% DMSO in liquid nitrogen. BAL wash fluid (4 × 10 mL washes of PBS) was pelleted and an aliquot of wash fluid (15 mL) was cryopreserved. The remaining cells were resuspended into ELISpot media (RPMI 1640, 10% heat-inactivated human albumin, 1% L-glutamine, and 1% HEPES) and counted. BAL cells were then divided into their appropriate flow cytometry assay depending on cell yield.

### Multiparameter flow cytometry

Longitudinal BAL and PBMC samples were stained for leukocyte composition (phenotype) or antigen-specific T cell responses. In general, BAL cells were stained immediately and PBMC were cryopreserved and batch-analyzed at the end of the study.

For BAL analyses, cells were counted and aliquoted into a 96-well plate for either phenotype or intracellular cytokine responses (1 × 10^6^ cells/well). In cases where total BAL cell counts were low, cells were prioritized for the 14-hour stimulation assay. For the phenotype panel, cells were reconstituted in 500 nM dasatinib in ELISpot media to improve MR-1 (5-OP-RU; BV421) tetramer staining. Cells were incubated with MR1 tetramer (NIH Tetramer Core Facility, Atlanta, GA) for 30 minutes at 4°C. The MR1 technology was developed jointly by Dr. James McCluskey, Dr. Jaime Rossjohn, and Dr. David Fairlie, and the material was produced by the NIH Tetramer Core Facility as permitted to be distributed by the University of Melbourne. Cells were then washed and stained for viability (Zombie Live/Dead near IR; Invitrogen) for 10 minutes at room temperature. Cells were then washed, incubated with surface antibody cocktail for 20 minutes at 4°C, and fixed with 1% paraformaldehyde for 15 minutes. For the intracellular cytokine assay, cells were aliquoted (1 × 10^6^ cells/well) into either a media control well, purified protein derivative well (PPD; 20 μg/mL; AJ Vaccines), or ESAT-6/CFP-10 (1μg/mL each; BEI Resources). Stimulators were added and incubated for 2 hours, then brefeldin A (1μg/mL) was added for the remainder of the stimulation time (12 hours). Cells were washed and surface markers were stained similarly as described for the phenotype panel. However, after PFA fixation, cells were permeabilized with BD Cytofix/Cytoperm^™^ (BD; Cat No. 554714) for 10 minutes at room temperature. Intracellular markers were stained for 20 minutes at room temperature. Stained BAL cells were run within two days of staining.

Flow cytometry of BAL was performed using a Cytek Aurora (BD). FCS files were analyzed using FlowJo software for Macintosh (version 10.1). Frequencies relative to live leukocytes were used to calculate cell counts per BAL collection (Figure 2). In brief, cell counts enumerated on a hemacytometer were multiplied the frequency of the cell population of interest (e.g., T cells) relative to live leukocytes. BAL samples with low viability (< 70%) or elevated background autofluorescence were excluded from analyses. Lastly, due to the COVID-19 pandemic, several BAL collections were not collected to ensure the health and safety of our technical staff. Therefore, the number of BAL samples indicated in Figure 2 are reflective of samples collected prior to and over the course of the COVID-19 pandemic and met the inclusion criteria described above. Individual animal data are shown in Supplementary Data 1.

For PBMC analyses, cells were divided to be stained for phenotype or 14-hour stimulation as previously described^15^. PBMC stimulators include: H37Rv whole cell lysate (20μg/mL, BEI Resources) and ESAT-6/CFP-10 peptide pools (1μg/mL each). Cell were stained as follows: cells were stained with MR-1 tetramer for 20 minutes; washed twice with PBS/BSA (0.1%); then stained with viability dye for 20 minutes; washed twice with PBS/BSA (0.1%) and incubated with human FcR blocking reagent (Miltenyi); surface markers were stained for 20 minutes and washed three times with PBS/BSA (0.1%); cells were permeabilized with BD Cytofix/Cytoperm^™^ (BD; Cat No. 554714) for 20 minutes and intracellular markers were stained for 30 minutes. Data was acquired on a modified BD FACS Symphony A5 and analyzed in FlowJo software for Macintosh (version 10.8.1). Gating strategies are shown in Supplementary Data 5–8. All cytokine data presented are background-subtracted. Antibodies used in BAL and PBMC panels are listed in Supplementary Table 1.

### ELISPOT

ELISpot was performed as previously described^15^. In brief, 96-well plates were coated with monkey IFNγ antibody (clone MT126L, 15μg/mL), incubated overnight at 4°C, and wells were washed with sterile 1X phosphate-buffered saline (1X PBS). Wells were then blocked for 2 hours at 37°C/5% CO_2_ with ELISpot media (RPMI 1640 + 10% human albumin + 1% L-glutamine + 1% HEPES) and washed with sterile 1X PBS. Frozen PBMCs were thawed and enumerated by hemocytometer. Cells were aliquoted into wells at 2 × 10^5^ cells/well (150μL). Stimulators or ELISpot media control (50μL) were added to the appropriate wells, and plates were incubated at 37°C/5% CO_2_ for 40 to 48 hours. The stimulators and the final concentrations used in this assay include: CFP (2μg/mL), ESAT-6 (2μg/mL), CFP-10 (2μg/mL), and phorbol 12,13-diburtyrate and ionomycin (12.5μg/mL and 37.5μg/mL, respectively). Wells were washed with 1X PBS and incubated with biotinylated anti-human IFNγ antibody (clone 7-B6, 2.5μg/mL) for 2 hours at 37°C/5% CO_2_. Wells were again washed with 1X PBS and incubated this time with streptavidinlinked horseradish peroxidase (HRP) (1:100 dilution in 1X PBS + 0.5% fetal bovine serum) for 45 minutes at 37°C/5% CO_2_. After washing with 1X PBS for a third time, 3-amino-9-ethylcarbazole (AEC) substrate was added to the wells and the plate was developed for 5 to 8 minutes in the dark (AEC kit). Finally, wells were washed with diH2O, fixed with 2% paraformaldehyde for 10 minutes, washed with 1X PBS, and dried overnight. Spot forming units (SFU) were counted manually on an ELISpot reader.

### Serology

The antibody ELISAs were performed as previously described by Darrah et al 2020. MaxiSorp ELISA plates (96-wells; Thermo Scientific Nunc) were coated with 100μL of H37Rv whole cell lysate (WCL) at a concentration of 1μg/mL per well at 4°C overnight. The coated plates were blocked with 100μL of 1X blocking solution (PBS+10% FBS) for 2 hours at room temperature. Plates were washed six times with PBS-Tween between each step. Plasma or 10X BAL concentrate from each animal were 1:5 serially diluted. 100μL was added and incubated at 37°C for 2 hours. After 2 hours, plates were incubated with 100μL of diluted HRP conjugated antibody as detailed in the table below. Plates were incubated for 1 hour at room temperature. A final wash step was done, samples were incubated for approximately 12 minutes with 100μL Ultra TMB ELISA substrate (Invitrogen, cat. no. 34029). The reaction was stopped by adding 100μL of 2N sulfuric acid. The plates were read with a Promega Glomax Multi detection system at 450 nm. Analysis was done using GraphPad Prism software. Data is presented as area under the curve (AUC).

### PET/CT

Radiolabeled 2-deoxy-2-(^18^F)fluoro-D-glucose (FDG) PET/CT imaging was performed 4, 8, and 12 weeks after Mtb challenge. Imaging was performed using a MultiScan LFER-150 PET/CT scanner (Mediso Medical Imaging Systems, Budapest, Hungary) housed within our BSL3 facility as previously described^57,58^. Co-registered PET/CT images were analyzed using OsiriX MD software (version 12.5.2, Pixmeo, Geneva, Switzerland) to enumerate granulomas and to calculate the total FDG avidity of the lungs, exclusive of lymph nodes, which is a quantitative measure of total inflammation in the lungs^57,59^. For historical controls, PET/CT scans were performed using a microPET Focus 220 preclinical PET scanner (Siemens Molecular Solutions) and a clinical eight-slice helical CT scanner (NeuroLogica Corporation)^59^. Thoracic lymphadenopathy and extrapulmonary dissemination of Mtb to the spleen and/or liver were also assessed qualitatively on these scans.

### Necropsy, pathology, and bacterial load

Necropsies were performed at around 12 weeks following Mtb challenge as previously described^19,20^ We used an established scoring system to quantitatively assess gross pathology^18^. Pathology scores were calculated and reflect overall TB disease burden for each animal. Tissue samples were divided and a portion was fixed in 10% neutral buffered formalin (NBF) for histopathology; the remainder was homogenized to a single-cell suspension as described previously^18^. Serial dilutions of these homogenates were plated onto 7H11 agar, incubated at 37°C, 5% CO_2_ for three weeks, and colonies were enumerated. Bacterial burden in lungs, thoracic lymph nodes, liver, and spleen, as well as total thoracic CFU, were calculated as described previously^18^. NBF-fixed tissue was embedded in paraffin, sectioned, and stained with hematoxylin and eosin for histopathologic examination.

### Statistics, inc. power analysis

We used total thoracic bacterial burden (log_10_) as the primary outcome variable with a pooled standard deviation of 1.09 (calculated by averaging standard deviations of all groups) for a two-sided test and adjusted the type I error for two comparisons (alpha = 0.025). For the unvaccinated (n = 8) and IV BCG (n = 7) comparison, we obtained 83.0% power to detect a mean difference of 2 in bacterial burden. For the SIV/Unvaccinated (n = 11) and SIV/IV BCG (n = 12) comparison, we obtained 97.0% power to detect a mean difference of 2. Normality was tested by using Shapiro Wilk test. Significance of plasma viremia relative to BCG within SIV+ groups was assessed using repeated measure one-way ANOVAs paired with Dunnett’s multiple comparisons tests to assess significance relative to ‘pre BCG’. For all other longitudinal comparisons between vaccinated groups, linear mixed effects models with subject as a random variable were used (Supplementary Table 2). Fixed effect tests were used to assess differences among time points and vaccine groups (including an interaction term of time x vaccine). Time points were compared to ‘pre BCG’ control using Dunnett’s multiple comparison tests. When a treatment effect was present (*i.e*. SIV infection), we performed Mann-Whitney tests at individual time point (not adjusting for multiple comparisons). For comparisons among all four groups across specific timepoints (e.g. pre vs post Mtb), linear mixed effects models with Šidák’s multiple comparisons were used (Supplementary Table 2). Comparisons between pre and post (relative to either BCG or Mtb challenge) were tested using either paired t tests or Wilcoxon paired signed rank tests, depending on normality. Kruskal Wallis tests were performed with Dunn’s multiple comparisons between SIV-naïve, vaccinated and unvaccinated groups, and SIV+, vaccinated and unvaccinated groups for necropsy outcome data (*e.g*. lung inflammation, TB pathology, and Mtb burden). Statistical tests were performed in Prism (version 8.2.1; GraphPad). All tests were two-sided, and statistical significance was designated at a *P* value of < 0.05.

## Extended Data

## Data Availability

All relevant data are available from the corresponding author upon reasonable request.
